# Journal of Ovarian Research reviewer acknowledgement 2012

**DOI:** 10.1186/1757-2215-6-16

**Published:** 2013-04-16

**Authors:** David T Curiel, Sham S Kakar, Stefano Palomba, Benjamin K Tsang

**Affiliations:** 1 Cancer Biology Division, School of Medicine, Washington University in St. Louis, St. Louis MO 63108, USA; 2 Department of Physiology and Biophysics, University of Louisville, Louisville KY 40202, USA; 3 Department of Obstetrics, Gynecology and Pediatrics, Sterility Centre P. Bertocchi, Obstetrics and Gynecology Unit, A.O. S.Maria Nuova, IRCCS, Reggio Emilia and University of Modena, Reggio Emilia, Italy; 4 Department of Obstetrics & Gynaecology, University of Ottawa, Ottawa K1H 8L6, Canada

## Abstract

**Contributing reviewers:**

The editors of *Journal of Ovarian Research* would like to thank all of our reviewers who have contributed to the journal in volume 5 (2012).

## 

David Abbott

United States of America

Nermeen Abdel Fattah

Egypt

Nuzhat Ahmed

Australia

Adriana Albini

Italy

Winfried Amoaku

United Kingdom

Satoshi Anai

Japan

Ritu Aneja

United States of America

Sharmila Bapat

India

Susan Bellis

United States of America

Giuseppe Benagiano

Switzerland

Deepa Bhartiya

India

James Derek Brenton

United Kingdom

Molly Brewer

United States of America

Leif Bungum

Sweden

Ralf Butzow

Finland

Karen Chan

Hong Kong

Subhash Chauhan

United States of America

David Covell

United States of America

Costantino Di Carlo

Italy

Fabio Facchinetti

Italy

Angela Falbo

Italy

Warren Foster

Canada

Luca Gianaroli

Italy

Digant Gupta

United States of America

Matthew Hall

United States of America

Chi-Chen Hong

United States of America

Patricia Hoyer

United States of America

HeFeng Huang

China

Kaisa Huhtinen

Finland

Venkatakrishna Jala

United States of America

Kannamannadiar Jayaprakasan

United Kingdom

Selvendiran Karuppaiyah

United States of America

Hidetaka Katabuchi

Japan

Jaehoon Kim

South Korea

Terumi Kohwi-Shigematsu

United States of America

Ikuo Konishi

Japan

Pamela Kreeger

United States of America

Deepak Kumar

United States of America

Miki Kushima

Japan

Antonio La Marca

Italy

Giovanni Battista La Sala

Italy

Hernan Lara

Chile

Djuro Macut

Serbia

Antonis Makrigiannakis

Greece

Massimo Manno

Italy

Helen Mason

United Kingdom

Richard Moore

United States of America

Kenneth Nephew

United States of America

Ceana Nezhat

United States of America

Francesco Orio

Italy

Sandra Orsulic

United States of America

Raoul Orvieto

Israel

Siva Kumar Panguluri

United States of America

Manish Patankar

United States of America

Chun Peng

Canada

Christine Ploger

Brazil

Meeta Pradhan

United Kingdom

Eric Prossnitz

United States of America

Jie Qiao

China

Sérgio R Soares

Spain

Tilman Rachner

Germany

Maria Rosaria Raspollini

Italy

Mariusz Ratajczak

United States of America

Suman Rice

United Kingdom

Joanne Richards

United States of America

Rajeev Samant

United States of America

Dorota Sanocka

Poland

Xavier Santamaria

Spain

Jung Ho Shin

South Korea

Maria Shoshan

Sweden

Makio Shozu

Japan

Viji Shridhar

United States of America

Yimin Shu

United States of America

Ajay Singh

United States of America

Edgardo Somigliana

Italy

Poli Mara Spritzer

Brazil

Oleh Stasyk

Ukraine

Xiao-Feng Sun

Sweden

Oya Topaloglu

Turkey

Fabian Trillsch

Germany

Swamy Tripurani

United States of America

Toshio Tsubota

Japan

Irma Virant-Klun

Slovenia

Weihua Wang

United States of America

Xuefeng Xia

China

Li Yali

China

Pumin Zhang

United States of America

Xiaodong Zhou

United States of America

